# Alzheimer's disease: part 2 – the present

**DOI:** 10.1055/s-0044-1791755

**Published:** 2024-11-11

**Authors:** Ricardo Nitrini

**Affiliations:** 1 Universidade de São Paulo, Faculdade de Medicina, São Paulo SP, Brazil.

**Keywords:** Alzheimer Disease, Biomarkers, Cholinesterase Inhibitors, Memantine, Neuroimaging, Antibodies, Monoclonal, Doença de Alzheimer, Biomarcadores, Inibidores da Colinesterase, Memantina, Neuroimagem, Anticorpos Monoclonais

## Abstract

Based on my work as a clinical neurologist with more than 50 years of experience in caring for patients with Alzheimer's disease (AD), I focus, in this review article, on the disease's two fundamental aspects for the doctor: diagnosis and treatment. The 1984 diagnostic criteria had been stable for more than a quarter of a century when it was replaced in 2011. Since then, there have been many discoveries, especially of biomarkers that have a heavy impact on the diagnosis of AD. Recently, AD biomarkers have become available in plasma, which certainly will cause a major change in the diagnosis of biological AD, a term that still needs care and information to society before being used in clinical practice. Three monoclonal antibodies against β-amyloid peptide have also been recently approved, and two of these have shown a small but statistically significant effect on clinical outcome. These monoclonal antibodies have had a greater effect on the reduction of amyloid plaques in the brain assessed by positron emission tomography (PET), and on the concentration of biomarkers in the cerebrospinal fluid (β-amyloid peptide with 42 amino acids and hyperphosphorylated tau protein) than in the neuropsychological and functional assessments. Even this small clinical effect will be encouraging for the development of new research, particularly helped by the greater ease of diagnosis and monitoring of the evolution of AD pathophysiology with plasma biomarkers. Recently, new diagnostic criteria for AD were presented by the Alzheimer's Association, causing controversy about their use in clinical practice.

## Resumo

Com base na minha atividade de neurologista clínico com mais de 50 anos de experiência no atendimento de pacientes com doença de Alzheimer (DA), foco neste artigo de revisão nos dois aspectos fundamentais da doença para o médico: o diagnóstico e o tratamento. Os critérios diagnósticos de 1984 permaneceram estáveis por mais de um quarto de século quando foram substituídos em 2011. Desde então, muitas descobertas foram feitas, especialmente de biomarcadores que têm grande impacto no diagnóstico da DA. Recentemente, biomarcadores de DA tornaram-se disponíveis no plasma, o que certamente causará uma grande mudança no diagnóstico da DA biológica, termo que ainda necessita de cuidados e informações para a sociedade antes de ser utilizado na prática clínica. Três anticorpos monoclonais contra o peptídeo β-amiloide também foram aprovados recentemente e dois deles mostraram efeito pequeno, mas estatisticamente significativo, no resultado clínico. Esses anticorpos monoclonais tiveram maior efeito na redução de placas amiloides no cérebro avaliadas por tomografia por emissão de pósitrons ( *positron emission tomography* , PET, em inglês) e na concentração de biomarcadores no líquido cefalorraquidiano (peptídeo β-amiloide com 42 aminoácidos e proteína tau hiperfosforilada) do que nas avaliações neuropsicológicas e funcionais. Mesmo este pequeno efeito clínico poderá ser encorajador para o desenvolvimento de novas pesquisas, particularmente auxiliado pela maior facilidade de diagnóstico e monitoramento da evolução da fisiopatologia da DA com biomarcadores plasmáticos eficientes para diagnóstico ou controle evolutivo. Recentemente, novos critérios diagnósticos para a DA foram apresentados pela Alzheimer's Association causando controvérsia quanto ao seu emprego na prática clínica.

## INTRODUCTION

My perspective is that of a clinical neurologist with over 50 years of experience caring for patients with Alzheimer's disease (AD). Therefore, I will focus on two of the main aspects of clinical care: diagnosis and treatment.

## DIAGNOSIS

The diagnosis of AD has been predominantly clinical for much of the past 50 years. In the diagnostic criteria published in 1984, 1 usually referred to as National Institute of Neurological and Communicative Disorders and Stroke and the Alzheimer's Disease and Related Disorders Association (NINCDS-ADRDA) criteria, which prevailed until 2011, AD was mainly characterized by cognitive and functional decline beginning with a decline in episodic memory. The diagnosis was clinical, and complementary exams were used only to exclude other causes of dementia. It was possible to make the diagnosis of possible or probable AD with clinical and neuropsychological assessment, while the definitive diagnosis of AD (or definite AD) depended on a neuropathological study, as long as there was a compatible clinical picture; in other words, the association of a clinical diagnosis of AD dementia and a neuropathological diagnosis was necessary for the definitive diagnosis. 1

### Advances after the 1984's NINCDS-ADRDA criteria

Despite advances in research and clinical activity, the NINCDS-ADRDA criteria were maintained for more than a quarter of a century.

The information provided by neuroimaging methods, both magnetic resonance imaging (MRI) and fluorodeoxyglucose-PET (FDG-PET), has greatly increased the physician's diagnostic confidence. 2 3 4 There have also been improvements in brief cognitive tests performed by physicians 5 6 or in more comprehensive neuropsychological assessments, 7 with better distinction between pathological cognitive decline and those caused by aging 8 and the effects of low education on the performance in cognitive tests. 9 10

There was still a lot of diagnostic uncertainty, especially in the early stages, such as mild cognitive impairment due to AD, 11 when imaging tests may not yet demonstrate evident changes.

Based on the hypothesis that neuropathological changes in AD precede clinical changes by many years, as occurs in degenerative diseases, there has been research into biological markers (biomarkers) that could reveal the presence of pathological changes before clinical manifestations. The leading biomarker candidates were the 42 amino acids (aa) β-amyloid peptide and hyperphosphorylated tau protein, according to the pathophysiology of AD. Studies have shown that in the early stages of AD, long before the appearance of clinical manifestations, the concentration of the 42aa β-amyloid peptide is lower, 12 13 and that of the hyperphosphorylated tau protein is higher in the cerebrospinal fluid (CSF). 14 15

Although these discoveries about biomarkers in CSF were made a long time ago, there were many difficulties in using them, due to the lack of standardization causing poor reliability among different laboratories.

Another important biomarker used a derivative of a dye used in neuropathological studies—thioflavin—which preferentially binds to amyloid present in neuritic plaques. 16 When thioflavin T-derivative is associated with radioactive isotopes and injected into the bloodstream, this biomarker can be identified in the cerebral cortex by PET, characterizing a positive-amyloid PET exam. 16 The first compound used—Pittsburgh compound B (PiB)—used carbon (C ^11^ ), but it was soon verified that it is possible to use fluorine (F ^18^ ), which has slower radioactive decay, thus allowing transportation from the center where it is produced to the exam sites. 17

As knowledge expanded, there was much criticism regarding the lack of changes of the NINCDS-ADRDA criteria.

### Diagnostic recommendations from the National Institute on Aging -Alzheimer's Association workgroups on diagnostic guidelines for AD (2011)

The diagnoses of possible and probable AD were maintained, and the diagnosis of pathophysiological proved AD dementia applies if the patient meets the clinical and cognitive criteria for AD dementia and the neuropathological examination demonstrates the presence of the AD pathology. 18

Although admitting that the form that begins with difficulty with episodic memory (amnestic form) is the most common one, atypical forms of AD were admitted that may begin with progressive language disorder (progressive aphasia, more typically the logopenic form) or with visual and visuospatial disorders that characterize the syndrome called posterior cortical atrophy or with dysexecutive-behavioral syndrome, which is very similar to frontotemporal dementia. 18

There are many changes that have been adapted to what was already in use in clinical activity in many centers, such as the use of some injury biomarkers that are relevant to confirm the clinical diagnosis. The most used currently are brain MRI, which can reveal hippocampal atrophy and diffuse cortical atrophy or with parietal predominance; and FDG-PET, which reveals predominant cortical hypometabolism in the parietal and posterior temporal cortex, with involvement of the posterior portion of the cingulate gyrus and precuneus. 18

Additional results were admitted that increase the level of certainty for possible and probable AD dementia. These results were obtained using two classes of biomarkers:

- biomarkers of brain amyloid-β (Aβ) protein deposition: low CSF Aβ42 and positive PET amyloid imaging;
- biomarkers of downstream neuronal degeneration or injury: elevated CSF tau, both total tau and phosphorylated tau (p-tau); and brain MRI and FDG-PET as described above.

The use of more specific AD biomarkers such as low CSF Aβ42, high CSF p-tau and positive PET amyloid imaging allowed the inclusion of two new categories: probable AD dementia with evidence of the AD pathophysiological process, and possible AD dementia with evidence of the AD pathophysiological process. 18

Biomarker evidence was also integrated into the diagnostic formulations for probable and possible AD dementia for use only in research settings. Several factors opposed inclusion in the clinical setting, including the unavailability for employment in most communities and the limited standardization of biomarkers from one locale to another. But their use was also proposed as “optional clinical tools when deemed appropriate by the clinician.” 18

### The amyloid, tau, neurodegeneration (ATN) framework

After 2011, there were other important advances in diagnosing the presence of AD pathophysiology. Highlights include the development of tau-PET, which marks the presence of the hyperphosphorylated tau protein present in neurofibrillary tangles (NFTs) in brain tissue 19 20 and advances in preanalytical and analytical methods for analyzing biomarkers in CSF, which have greatly increased the reliability of these methods. In 2016, the ATN framework was proposed, a set of data that allows the diagnosis of the pathophysiology of AD to be used in research based on the hypothesis that treatment would be more successful if started very early and on the findings that many patients included in clinical trials for the treatment of AD did not have AD, especially when in the early stages. 21

With the aid of complementary tests that demonstrated low concentration of β-amyloid peptide of 42aa in the CSF and positive amyloid PET ( **A** ), an increase in the concentration of hyperphosphorylated tau protein in the CSF and tau-PET( **T** ), associated with neurodegeneration ( **N** ) the diagnosis of the pathophysiology of AD could be performed. Proof of N can be performed with structural neuroimaging exams such as computed tomography and especially MRI and/or FDG-PET; and increased concentration of total tau protein in CSF. 21

The ATN framework was defined independently of the clinical picture, 21 as its objective was to research the presence of AD pathophysiology. The main principles of the framework were presented with more details in 2018. 22 Despite the recommendation for use only in research, the use of biomarkers in CSF also began to be used in clinical practice for cases of dementia that were more difficult to diagnose.

However, these diagnostic methods had the main disadvantages of high costs, the lack of laboratories prepared for the diagnosis of biomarkers in the CSF, and the low availability of equipment and professionals trained to analyze the results of amyloid PET and tau-PET. 21 22

### Plasma biomarkers

Research into AD biomarkers in plasma has developed significantly in recent years with technical improvements and evidence that they can be used in research, 23 24 25 and, even more recently, in clinical activity. 24 26 The concentrations of β-amyloid peptide of 42aa and hyperphosphorylated tau protein, especially when phosphorylated in sites indicative of AD, such as p-tau217, 25 p-tau181, and p-tau231, can be used for research and also in clinical practice, although doubts still remain as to whether there is a need for confirmation with other biomarkers. 27 28

Other biomarkers indicative of brain injury such as neurofilament light chain (NfL), brain-derived tau, and glial fibrillary acidic protein (GFAP) have been proposed. They are not specific for AD but may provide information about the evolution of the disease. 27 28

However, plasma biomarkers still require many advances in knowledge and technical improvements so that they should not be used as stand-alone tests in clinics (there are still questions about many preanalytical care, measurement techniques, established thresholds, and harmonization of care with samples to allow comparison between different results). 27

The ease of requesting plasma tests to search for AD biomarkers can cause some problems. First of all, it is important to highlight that these tests should not be requested for patients without symptoms of cognitive decline. 29 30 31 The condition called subjective cognitive decline (SCD), in which there are complaints of decline, but the objective assessment of cognition is normal, 32 has raised doubts regarding the indication of requesting plasma biomarkers. 29 30 31

Some issues related to plasma biomarker research must be demonstrated and discussed with patients as well as explained to doctors who inadvertently order these tests. There are still preanalytical problems regarding the best collection and storage procedures, doubts regarding the presence of comorbidities and use of medications, and the thresholds are not well established. 27 The results must be confirmed, when necessary, by researching biomarkers in the CSF or amyloid PET and/or tau PET. 29

### Biological AD

In the Alzheimer Association International Conference, in July 2023, the diagnosis of biological AD was proposed to be used in clinical practice. 33 According to this proposal, the presence of AD pathophysiology identified by biomarkers in CSF, neuroimaging (amyloid PET and/or tau PET), and/or plasma biomarkers, would allow the diagnosis of “biological AD,” when there are suggestive clinical signs or even in their absence. The term “biological AD” (or pathophysiology of AD) would enter into clinical practice. 33

According to that proposal, biomarker abnormalities of β-amyloid protein and phospho-tau protein in the CSF, or neuroimaging or plasma, should be diagnosed as AD or as “biological AD,” even in the absence of any clinical symptom. 33

This proposal was submitted to experts' criticisms and suggestions by the Alzheimer's Association.

There was strong opposition to the use of this concept in clinical practice, particularly from the International Working Group, which holds that “biomarker-positive cognitively unimpaired individuals should be considered only at-risk for progression to AD.” 31

In October 2023, due to criticisms and suggestions, the Alzheimer Association Working Group slightly modified its proposal. 34

According to the Revised Criteria for Diagnosis and Staging of Alzheimer's Disease: Alzheimer Association Workgroup, it was clearly stated that these proposals “are not intended to be specific clinical practice guidelines, but rather criteria to inform diagnosis and staging of AD that reflects current science.” It was emphasized that routine diagnostic testing is not recommended for asymptomatic individuals, at this time. 34

In August 2024, the Revised Criteria for Diagnosis and Staging of Alzheimer's disease: Alzheimer Association Workgroup was published. 35 The main aspect of these criteria is of a staging system for sporadic AD is that it takes into account the 6 clinical stages of the disease from asymptomatic (1) to severe dementia (6), and the 4 biological stages that are defined by amyloid PET and tau PET. In the initial biological stage, only amyloid PET is positive (A), whereas in the early (B), intermediate (C), and advanced (D) stages, tau-PET is also positive, but involving only the medial temporal lobe in B, then also moderate neocortical uptake in C, and high neocortical uptake in D. With the clinical and biological stages is possible to classify AD cases as 3B or 4C or 6D. This model follows the classification that has been used for classifying diseases in oncology. 35

The main limitation of this staging system is the need of tau-PET, 35 which is not available in most centers.

### Biological AD in research

It is unnecessary to discuss how important the concept of “biological AD” will be for research. From diagnostic certainty and the ease of finding cases in the initial phase for clinical trials as well as obtaining dynamic biomarkers that can be verified to evaluate and investigate the effectiveness of the treatment, such as PCR in acute infections, blood glucose in diabetes, and the viral load in HIV, for example. Examples of these biomarkers that are in research for monitoring AD response to treatments are concentrations of NfL and GFAP, which could be also used as risk markers for conversion to AD dementia. 27 28 Although they are not specific to AD, they reflect the severity of degeneration, as explained above.

### Biological AD in the clinical practice

The positivity of these biomarkers is a risk factor for the development of AD dementia.

As patients with SCD are increasingly turning to neurologists and other specialists for diagnosis and treatment, and want to investigate the possibility of AD, some information is important when caring for these patients.

The first is that biomarker positivity is low in SCD, that is, in most cases it is not due to AD.

In 1 study, among 693 subjects (41% women), 60 ± 9 years, with SCD, the mean Mini-Mental State Examination (MMSE) score of 28 ± 2, only 122 (17.6%) had positive CSF biomarkers for AD. 36

In another study that included 1,325 individuals (who were older than those in the above-mentioned study), 37 843 (63.6%) had no amyloid and tau PET abnormalities, 328 (24.7%) were only amyloid positive, while 120 (9.0%) were positive for both amyloid and tau PET. (In 34 cases, participants were only tau-PET-positive). 37 Even among those 120, who were positive for both amyloid and Tau PET, more than 40% remained cognitively normal after an average period of 3 and a half years of follow-up. 37 Therefore, they did not progress to dementia or even to mild cognitive impairment (MCI), demonstrating that there are more resilient individuals. 37

The most important question here concerns the meaning of AD. For the last almost 50 years, we have explained to the population that AD is the main cause of dementia and a “major killer.” 37 Alzheimer's disease was presented to the world population as a synonym of dementia, and this is how it is still interpreted by the media. Now we would use almost the same term, “biological AD” for a completely different situation. The adjective *biological* will not change much of the lay public's understanding. A major campaign will be needed to explain the meaning of “biological AD” to society as a whole. And that an individual with “biological AD” cannot be passed over for any position they propose to occupy. 38

## Treatment

In this article, we will focus on pharmacological treatment, but it is necessary to say that there is evidence that there are measures to prevent cognitive decline and dementia that occur in aging, of which AD is the main cause.

### Preventive treatment

One of the main confirmations of success with preventive treatment of dementia was obtained from a study performed in Finland, known as Finger, which included individuals aged 60 to 77 years with mild cardiovascular risk factors and with cognition at mean level or slightly lower than expected for age. 39 The multidomain intervention lasting 2 years consisted of diet, exercise, cognitive training, and vascular risk monitoring. Individuals were randomly selected to participate in the intervention group, or in the control group that received general health advice. It was possible to verify that there was less cognitive decline in the group undergoing multidomain intervention. The difference was small but statistically significant. 40 This study is being replicated in many countries around the world. 41 Two Brazilian universities, Universidade de São Paulo and Universidade Federal de Minas Gerais, take part in the study called LatAM-Finger, in which 12 Latin American countries participate. 41

It should be noted that this study aimed to verify whether the multidomain intervention reduced decline and was not intended to specifically assess decline caused by AD, and knowing that the effect of the intervention was greater in individuals who were at higher risk of cardiovascular risk and were older. 39

### Risk factors for dementia

A well-known study showed that there are 12 potentially modifiable risk factors for dementia, and that their control could reduce the incidence of dementia in the elderly by 40%. 42 The most important are low education, high blood pressure, diabetes mellitus, trauma, cranial disorders, alcoholism, sedentary lifestyle, obesity, in addition to untreated depression, social isolation, hypoacusis, air pollution, and smoking. 42 In Brazil, due to socioeconomic factors, such as very low education and nutrition problems in childhood, control of these factors risk could further reduce the incidence of dementia, reaching 48%. 43 More recently, two other potentially modifiable risk factors (untreated vision loss and high blood LDL cholesterol) were added to the list. 44

Alzheimer's disease is the leading cause of dementia in the elderly, but not the only one. Recent studies have demonstrated that in neuropathological studies in which cases with a clinical diagnosis of AD were evaluated, less than 20% had AD alone. 45 The majority of cases had AD associated with other diseases, such as Lewy body disease, cerebrovascular disease, cerebral amyloid angiopathy, hippocampal sclerosis, and other types of pathological changes. 45 The frequency of multiple diagnoses was also more frequent in the very old compared with younger participants. 46 This information is important to understand why the effect of the Finger preventive intervention was greater in individuals who were at higher cardiovascular risk and were older in the study. 39

### A counterpoint

It is clear that more than 50% of the risk factors for dementia (and AD) are currently not controllable.

It is accepted in the literature that heritability for AD is ∼ 60 to 80%. 47 48

In both types of studies, epidemiologic and genetic, no careful distinction is usually made between AD and other forms of dementia that are common in aging, often associated with AD.

In any case, genetic factors are most likely more important than environmental ones. And, as we still do not have prevention for them, they will be presented in more detail in the third part of this article.

### Pharmacological treatment

The usual treatment is based on the discovery of reduced acetylcholine activity in the cerebral cortex of patients with AD. Many paths led to this discovery. Perhaps the first was based on the observation of the side effects of anticholinergic medications, which are memory and attention impairments (anticholinergic drugs are currently not recommended for use in the elderly and in patients with cognitive decline), 49 or the finding that the use of anticholinergics, such as scopolamine, subcutaneously in young individuals caused memory disorders similar to those observed in patients with AD. 50 Then, there was the observation of a reduction in the number of cholinergic neurons in the nuclei of the basal forebrain, mainly in the nucleus basalis of Meynert (for a review of the topic, see Mesulam). 51 Axons with origin in cells of these nuclei have modulatory effects on broad areas of the cerebral cortex, having acetylcholine as a neurotransmitter. Attempts to use acetylcholine precursors in AD were unsuccessful and an acetyl cholinesterase inhibitor (ChEI) with an effect on the central nervous system—tetrahydroaminoacridine—improved the performance of AD patients on memory tests. 52 That article was welcomed by an editorial, because, for the first time, there was a treatment for AD based on scientific observations and research. 52 53

Tetrahydroaminoacridine (tacrine) had hepatotoxic effects and other ChEIs replaced it. Donepezil, rivastigmine, and galantamine are the main ChEIs we use today to treat AD symptoms. These three drugs have a similar clinical effect, causing a slight improvement in memory and attention and have similar side effects that depend on the stimulation of peripheral muscarinic receptors. 54

The treatment effect is small and does not modify the pathophysiology of the disease and the clinical evolution. In other words, they are classified as drugs with a symptomatic effect.

The usual doses of the ChEIs are in Table 1 .

**Table 1:** Usual doses of acetylcholinesterase inhibitors (ChEIs)

Drugs	Starting dose	Therapeutic dose	Router for administering
Donepezil	5 mg/day	5–10 mg/day	1 tablet once a day orally
Rivastigmine	3 mg/day	6–12 mg/day	1 tablet twice a day orally
Rivastigmine	4.6 mg/day	4.6–13.3 mg/day	Patch, once a day
Galantamine*	8 mg/day	16–24 mg/day	1 tablet once a day orally

Note: *Extended release.

A retrospective study performed in Italy compared AD patients who received ChEIs with those who did not. 55 It was shown that treatment with ChEIs was associated with a slower cognitive decline and with reduced mortality, after a mean follow-up of almost 8 years. 55

Another recent retrospective study performed in Sweden included a large number of AD patients who were treated with ChEIs and were compared with nonusers after 5 years. 56 The evolution of cognitive decline assessed by the MMSE, the evolution to severe dementia (MMSE score < 10), and death as outcome were investigated. Cholinesterase inhibitors use was associated with higher MMSE score at each visit and lower risk of death compared with nonusers. 56 The difference in this study was modest but significant. 56 Both studies provide class III evidence. 55 56

Another drug, memantine, acts as a non-competitive blocker of the N-methyl-D aspartate receptor and prevents the neurotoxicity of high concentrations of glutamate that occur in the moderate and advanced phases of AD. 57

Memantine (10 mg tablets, twice a day) was approved for use in moderate and advanced AD by the European Medicine Agency in 2002 and by the United States Food and Drug Administration (FDA) in 2003. 58 The association with donepezil has had a proven positive effect 59 and can prolong survival compared with the isolated use of donepezil or memantine use of neither. 60

Cholinesterase inhibitors and memantine have been the only specific pharmacologic treatments approved for AD dementia until recently.

The only drugs that were launched specifically for AD are mentioned here, but other drugs can be used for neuropsychiatric manifestations that will not be discussed here, neither will I discuss non-pharmacological treatments.

### Recently approved treatments

Three drugs that contain monoclonal antibodies against the 42aa β-amyloid peptide were approved by the FDA: aducanumab, lecanemab, and donanemab. The first was aducanumab, which had demonstrated an effect on reducing β-amyloid protein deposits with amyloid PET, and for which there was a strong expectation of success. 61 In two clinical trials performed, the primary outcome measure was defined as a change from baseline to week 78 on the Clinical Dementia Rating Sum of Boxes (CDR-SB. 62 63 The primary endpoint was met in only one of the two clinical trials. 62 63 Aducanumab treatment produced statistically significant, dose-dependent reductions in CSF p-Tau levels, CSF total-Tau levels, and in brain amyloid plaque versus placebo, as measured by PET, at week 78. 63 The approval of aducanumab by the FDA caused controversy throughout the scientific community because the clinical effect was small and had only been demonstrated in one of the two trials performed, which indicates that a third clinical trial should have been performed. 64 65 Aducanumab had its production suspended by Biogen at the beginning of 2024. 66

In July 2023, the FDA approved lecanemab, a medication that contains a monoclonal antibody against the 42aa β-amyloid peptide, 67 which binds with high affinity to soluble Aβ monomers, oligomers, and protofibrils. 67 68 As with aducanumab, it was possible to demonstrate a reduction in β-amyloid protein deposits with amyloid PET. 69

However, the size of the clinical effect, also assessed by CDR-SB, was small. Scores on this scale range from 0 (absolutely normal) to 18 points (severely compromised). At the beginning of the treatment, the average score in both groups was 3.18. After 18 months, the lecanemab-treated group had an average increase in CDR-SB of 1.21 while the placebo group had an increase of 1.66. A very small but statistically significant difference. 69 Furthermore, side effects were not infrequent, generally with the most common adverse events being infusion-related reactions, amyloid-related imaging abnormalities (ARIAs), which may present as edema (ARIA- E) or as hemorrhage (ARIA-H) and can be serious, 68 69 and which also had occurred with aducanumab. 63 64 70 Amyloid-related imaging abnormalities are more frequent when the patient is a carrier of the allele e4 of the *APOE* gene, mainly when it is in homozygosity. 71

Even the small effect of lecanemab may be considered as a proof of concept, demonstrating that reducing amyloid in the brain parenchyma has an effect on cognition. 71 This suggests that if treatment is started very early, it could prevent the progression of AD. 71 However, this is still a hypothesis, without any scientific evidence.

For others, the small magnitude of the effect proves that this is not the way to go. 72 Or this treatment will be associated with other more effective drugs (and with less side effects) in the near future. 69 The treatment requires intravenous infusion every 15 days, there are potentially ominous side effects, it is expensive and must have periodic control with brain MRI. 69 And it should not be misunderstood as a treatment for AD dementia, but as a treatment for the early stages of AD. 68 69 Important agencies, such as the European Medicines Agency and the National Institute for Health and Care Excellence (NICE) from the United Kingdom have not approved or are still evaluating lecanemab for clinical use. 73 74 Lecanemab has not yet been evaluated for approval by the Brazilian Health Regulatory Agency (Agência Nacional de Vigilância Sanitária [ANVISA], in Portuguese).

In July 2024, a new monoclonal antibody against 42aa β-amyloid peptide, donanemab, was approved by the FDA. 75 The TRAILBLAZER-ALZ 2 Randomized Clinical Trial showed that after 76 weeks of treatment, there was an impressive reduction of amyloid deposits, evaluated by amyloid PET. 76 The primary outcome of the study was a change on a scale integrating cognitive and functional evolution (integrated AD Rating Scale-[iADRS]). There was a statistically significant effect between donanemab and placebo on this scale, but it did not reach the minimal clinically important difference. In the study, it is informed that the meaningful within-patient change should be from 5 (for patients with MCI) to 9 points (for patients with mild AD) on the iADRS. 76 The mean observed differences in the study did not reach 4 points in either very mild MCI or mild AD. Other cognitive scales included as secondary outcomes did not reach significant differences. Otherwise, there was no difference between donanemab and placebo in tau PET change, which was included as a secondary outcome. 76 This is now a relevant issue, because tau PET is the biomarker for staging AD according to the new proposed criteria. 35

### Treatments under evaluation

In annual articles, the group led by Jeffrey Cummings publishes a list of drugs that are being researched for the treatment of AD. 77 The drugs are classified according to the main proposed mechanism of action, which ranges from acting on the pathophysiology (modifying effect on the disease) to a purely symptomatic effect; and also what phase of the study they are in. 77 At first glance, the number of drugs registered on clinicaltrials.gov is surprising.

In the last available publication (2024), there were 164 trials assessing 127 drugs, 32 of which are in phase 3 (i.e., in the presubmission phase for approval, if successful). 77 To analyze the current moment, it is interesting to focus on this group of 32 drugs, because they are the closest to being approved for the treatment of AD. Of these, 11 drugs targeted neurotransmitters/synaptic receptors, 7 the β-amyloid protein, 4 synaptic plasticity and neuroprotection; 2 drugs targeted neuroinflammation, 2 metabolism and bioenergy, and another 2 proteostasis and proteinopathies. Only 1 phase-3 study targeted tau protein, neurogenesis, growth factors, and circadian rhythm. 77

Most clinical trials are focused on the initial phase of AD, in which it is believed that it is possible to act to prevent progression and to evaluate cognitive enhancing agents that could have an effect on symptoms of cognitive decline in the MCI or dementia phase of AD. 77

Clinical trials in phases 1 or 2 are based on current ideas, and, if approved, they will become the treatments of the future. Therefore, they will be discussed in part 3 of this article, which will be about the future of AD.

### What would be the new paths?

Although there are already indications of paths to follow, they have not yet left the treatment bench, and I will save them for the part 3: the future.
